# Totally synthetic microperoxidase-11

**DOI:** 10.1098/rsos.172311

**Published:** 2018-05-23

**Authors:** Junichi Tanabe, Koji Nakano, Ryutaro Hirata, Toshiki Himeno, Ryoichi Ishimatsu, Toshihiko Imato, Hirotaka Okabe, Naoki Matsuda

**Affiliations:** 1 Department of Applied Chemistry, Faculty of Engineering, Kyushu University, 744 Motooka, Nishi-ku, Fukuoka 819-0395, Japan; 2 National Institute of Advanced Industrial Science and Technology, Kyushu, 807-1 Shuku-machi, Tosu, Saga 841-0052, Japan

**Keywords:** microperoxidase, total synthesis, high-speed atomic force microscopy, enzymatic assay, spectroelectrochemistry, electrocatalytic reaction

## Abstract

A totally synthetic microperoxidase-11 (MP-11) is reported. Accordingly, the undecapeptide (VQKCAQCHTVE) was synthesized by solid-phase peptide synthesis followed by the thiol-ene click reaction with haemin for reconstitution. High-speed atomic force microscopy measurement conducted in water confirmed the protein reconstitution by visualizing the morphological differences as animated molecular images. The synthetic MP-11 showed a considerable magnitude of catalytic activity (27%) against the natural MP-11 in the oxidation of 3,3′,5,5′-tetramethylbenzidine by hydrogen peroxide, whereas it showed very low (2.7%) activity of a synthetic variant with a point mutation (VQKCAQC***M***TVE, H8M). Slab waveguide spectroscopic measurements revealed that the ferrous/ferric redox reaction occurred by the direct electron transfer with specific spectral changes. Indeed, if hydrogen peroxide existed in the solution phase, the peroxidase-modified electrode showed catalytic current–voltage behaviour regardless of whether it was prepared using natural MP-11 or the synthetic MP-11. If a substrate recycling reaction was assumed, computer simulation well reproduced the experimental curves to give a global set of electrocatalytic reaction parameters. In any of the experiments, the synthetic MP-11 and natural MP-11 gave almost identical results. Our approach will be a convenient means of preparing MP-11, as well as its mutants, that does not rely on nature.

## 1. Background

Microperoxidases (MPs) have been establishing themselves as an attractive class of alternatives to haemperoxidases [1]. For example, a naturally occurring undecamer (VQKCAQCHTVE, MP-11) obtained proteolytically from cytochrome *c* contains a haem group that covalently bonds to the polypeptide chain via two thioester bonds of cysteine residues. Besides, the imidazole side chain of histidine binds to the haem iron to give the corresponding five-coordinate, high-spin complex. Even though the structure is minimal, MP-11 and its homologues show a substantial degree of peroxidase activity. Therefore, they have been widely used as models for haem active sites and for investigations of the oxidoreductase reaction mechanism [2–6]. MPs have further stirred intensive research for their use as oxidation catalysts for various chemical/biochemical purposes including biosensing [7–9], energy conversion [10,11] and biofuel cells [12].

Functional modification of MP-11 is also forming an interesting research field; for the oxidoreduction function not found in the original, demetalation of the Fe(III)-haem followed by complexation with Mn(III) [13] or Co(III) [14] has been examined. MPs with different amino acid sequences could be obtained from *Shewanella oneidensis* [15] or *Marinobacter hydrocarbonoclasticus* [16]. *In vivo* expression technology has produced various MP variants with peptide sequences that do not occur naturally [17]. Recently, Bren *et al*. extended to a biosynthesis method that does not rely on cytochromes *c* expression [18]. Working constantly on those naturally originating polypeptides, we can also expect any MP with an unusual function to be available.

While cytochromes occupy an important position as the raw material of MPs, solid-phase peptide synthesis (SPPS) is the primary tool for creating diverse polypeptides. As a result, synthetic methods are also yielding significant results; various peptide–porphyrin conjugates were studied and some of them achieved peroxidase-like activity, i.e. miniaturized metalloenzymes [19,20]. In addition to SPPS, highly substituted porphyrin derivatives contribute greatly by acting as covalently bound, catalytic sites. Currently though, installing of haem *c* (haemin) into host peptides has not been established.

**Figure 1.**
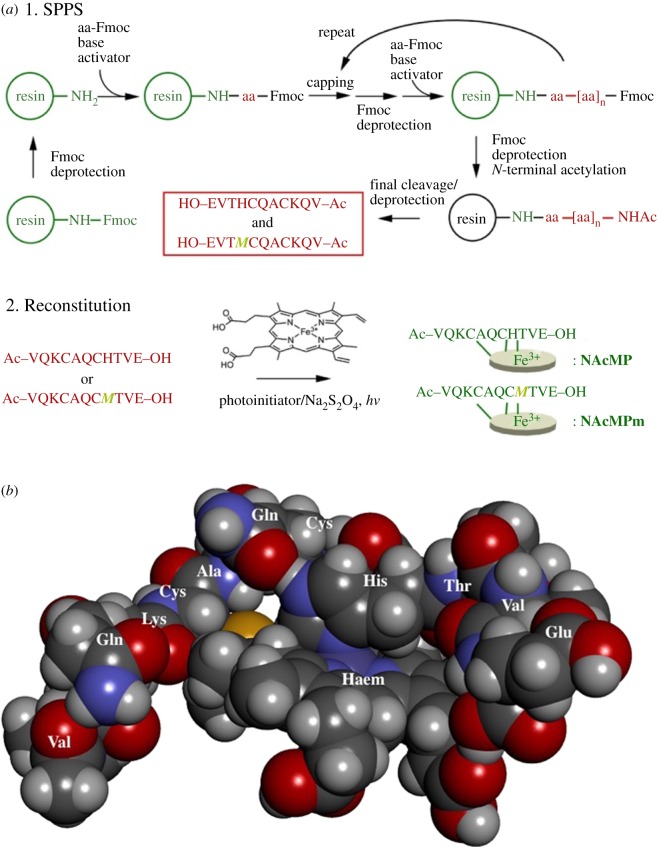
(*a*) The synthetic scheme for the initial SPPS of *N*-acetylated undecapeptides, followed by the thiol-ene click reaction for reconstitution. (*b*) A three-dimensional model of a theoretically optimized structure (Gaussian 09/LanL2DZ).

Here, we report a totally synthetic approach that yields MP-11; SPPS was used to synthesize the undecapeptide as the *N*-acetylated form, followed by the thiol-ene click reaction with haemin for reconstitution (**NAcMP**, figure 1). On the one hand, we expanded early research [21] by synthesizing an example of mutants, VQKCAQC***M***TVE (H8M); mitochondrial cytochromes *c* contain a *c*-type haeme with axial His18 and Met80 ligands and therefore, after reconstitution, H8M represents a homologous MP-11 having the opposite axial ligand other than His. On the other hand, we characterized the reconstitution reaction through single-molecule visualization using atomic force microscopy (AFM). Moreover, we collected information on the ferrous-to-ferric oxidoreduction reaction in detail, which becomes important in bioanalysis applications. In any of the experiments, results of the synthetic and the natural MP-11 were almost identical. Therefore, we concluded that our approach is a convenient means of MP-11 synthesis that does not rely on nature.

## 2. Experimental

### 2.1. Synthesis of MPs-11

Two-types of undecapeptide, VQKCAQCHTVE and VQKCAQC***M***TVE, were synthesized by Fmoc SPPS in a microwave synthesis system (Discover® SP, CEM Co., Matthews, NC, USA). The *N*-terminal of the final product was acetylated to improve the solubility and to prevent aggregation after being reconstituted to the holocompound [22]. The undecapeptides were further subjected to the thiol-ene click reaction [23] with haemin. The detailed synthetic procedures, including analytical data, and UV–visible spectra (electronic supplementary material, figures S1–S4) are provided as electronic supplementary material.

### 2.2. Atomic force microscopy imaging

AFM imaging was carried out using a high-speed atomic force microscope (Research Institute of Biomolecule Metrology (RIBM) Co., Ltd, Tsukuba, Japan). The cantilevers were typically silicon with a tip radius of 10 nm: length 10 µm, spring constant 0.1 N m^–1^, resonance frequency 1500 kHz. The sample peptides were dissolved in 2,2,2-trifluoroethanol, diluted with aqueous MgCl_2_ solution (1 mol dm^–3^) to give 0.5 mg ml^–1^ solution. Atop freshly cleaved mica was placed a 2 µl portion of the sample solution, left for 10 min at room temperature, and then the surface was rinsed with deionized water. A 100 µl portion of deionized water was placed on the sample surface and the surface topological images were continuously acquired at 10 frames per second. All AFM data thus obtained were processed and analysed by using a copy of Gwyddion [24]. All experiments were made at room temperature, typically 22 ± 2°C.

### 2.3. UV–visible study of the peroxidase reaction

An aliquot of **NAcMP** was dissolved in a phosphate buffer solution (0.01 M, pH 7.0). The UV–visible absorption at 406 nm (*A*_406_) of the solution was linearly dependent on **NAcMP** concentration in a concentration range investigated (0–36 µM, *r*^2^ = 0.999). Peptide concentrations were spectrophotometrically determined (59 µM, *ϵ*_406_ = 60 300 l mol^−1^ cm^−1^). A 3 ml portion of 0.143 mM 3,3′,5,5′-tetramethylbenzidine (TMBZ) was placed into a 1 cm quartz cuvette, then a required amount of haempeptide solution was added. The reaction was initiated by adding 1.5 µl of 0.1% H_2_O_2_ and the time course of TMBZ oxidation was measured by recording the absorbance at 655 nm (*ϵ*_max_ 5400 l mol^−1^ cm^−1^) [25]. Similarly, **NAcMPm** (58 µM) was also tested. The solution was gently agitated with a magnetic stirrer during measurement while the temperature was kept at 37°C.

### 2.4. Electrochemical and spectroelectrochemical measurements

Gold disc electrodes (ø1.6 mm) were used for cyclic voltammetry (CV) measurements in combination with a Pt-wire counter electrode. All electrode potentials were referenced to an Ag/AgCl electrode (3 M NaCl). For spectroelectrochemical measurements, a home-built slab optical waveguide (SOWG) spectroscopy system was used in combination with indium tin oxide (ITO) electrodes. A xenon lamp and a CCD equipped with a monochrometer were used as the light source and detector, respectively. For protein immobilization, each cleaned ITO electrode was treated with a 1.7 ml portion of **NAcMP** solution (103 µM in 0.01 M phosphate buffer, pH 7.0) for 30 min. Detailed experimental conditions were set according to a previous report [26]. For the direct electron transfer (DET) measurements, each cleaned Au electrode was immersed into a 10 mM ethanoic 6-mercapto-1-hexanethiol (HXT) solution for 12 h, and subsequently exposed to a 100 µl portion either of **NAcMP** or natural MP-11 solution for 30 min. A model ALS 750 potentiostat (BAS Inc., Tokyo, Japan) collected the electrochemical responses and its built-in software served to reproduce them. All electrochemical measurements were conducted at room temperature, typically 22 ± 2°C.

## 3. Results and discussion

### 3.1. Atomic force microscopy imaging in water

A previous report included the solution CD spectral data, which indicated that the undecapeptide became a somewhat developed secondary structure upon reconstitution: the apparent helical content improved from 13% to 14% [21]. However, the bulky prosthetic molecule haemin must have some influence on the higher-order structure of the host undecapeptide. High-speed AFM observation clearly depicted the structural changes by visualizing/animating the characteristic shape of each molecule.

**Figure 2.**
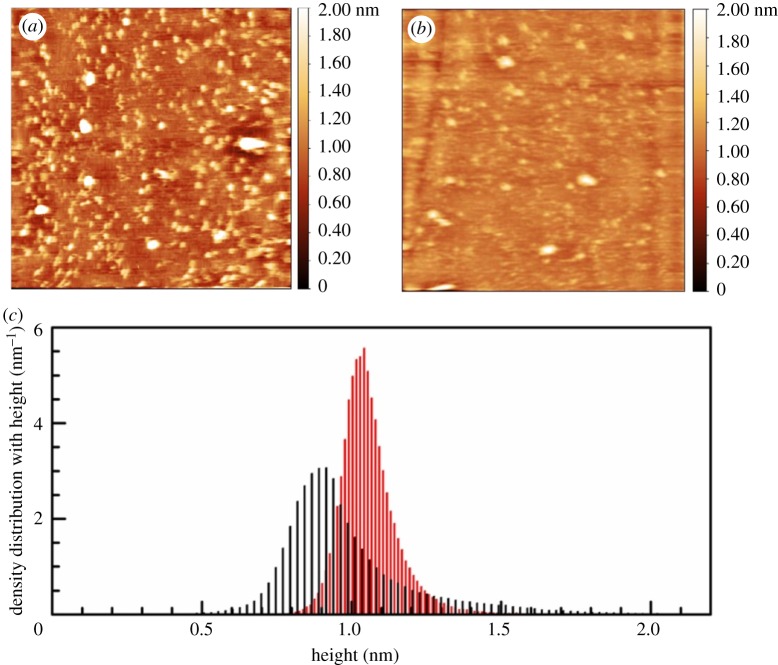
Representative AFM images (500 × 500 nm) for the undecapeptide (*a*) and **NAcMP** (*b*) obtained in water. (*c*) Plots of density distribution with height for the undecapeptide (black) and **NAcMP** (red).

As seen in figure 2, the surface of undecapeptide-treated mica sheet is covered with dozens of nanometre-sized substances. The apparent density became higher if larger amount of undecapeptide was used for sample preparations (data are not shown). Therefore, these surface bodies were expected to be undecapeptide molecules. Most of them have unique twisted coil shapes; it was about 6 nm in length and was 13 nm when it became extended. After reconstitution of haemin, most of the undecapeptide represented spherical morphology, typically 6 nm in diameter. The structural change was further evidenced by the density distribution with height. After reconstitution, the broad peak at 0.90 nm (FWMH 0.28 nm) for the undecapeptide shifts to larger direction with smaller FWMH, 1.04 nm and 0.17 nm, respectively, which evidences more compact spatial structure.

Protein structures fluctuate in conformational basins and, at the minima, are generally close to what X-ray analysis predicts. The fluctuation involves various molecular motions that exhibit a specific time scale ranging from sub-nanosecond to millisecond: the movement of the atomic groups, such as amides and methyl groups, is very fast, whereas large structural interconversions generally occur slowly in the microsecond to millisecond time scale. The animated AFM image (electronic supplementary material, figure S5) for the undecapeptide successfully visualized the temporal change of morphology. **NAcMP** is also active for the structural change. However, with the animated data one can notice that covalent binding of the prosthetic group markedly suppresses the conformational change of apopeptide chain.

### 3.2. Peroxidase activity towards oxidation of TMBZ

**Figure 3.**
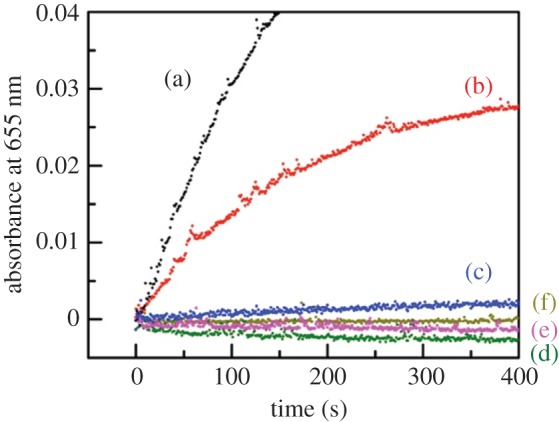
The time course of absorbance at 655 nm of 0.14 mM TMBZ solution (0.1 M phosphate buffer) in the presence of 15 µM H_2_O_2_ and each of MP-11 from cytochrome *c* (a), **NAcMP** (b), **NAcMPm** (c), apo-**NAcMP** (d), haemin (e), and without the catalytic component (f) at 37°C. The concentration of catalysis component was 0.68 µM (natural MP-11, **NAcMP** and **NAcMPm**) or 0.81 µM (apo-**NAcMP**).

Next, the peroxidase activity of **NAcMP** was examined by taking TMBZ oxidation in the presence of H_2_O_2_ as an example. Figure 3 compares the time course of the oxidation product; results clearly showed that **NAcMP** catalysed the reaction. A mixture including only TMBZ and H_2_O_2_ did not yield any product. Unsurprisingly, apo-**NAcMP** or haemin used alone was inactive. The specific activity of **NAcMP** towards TMBZ oxidation was 0.43 mol min^−1^ mg^−1^, which remained at 27% of natural MP-11 (1.6 mol min^−1^ mg^−1^). Catalytic reaction of haemperoxidase is explained by the ‘push–pull’ mechanism [27], which involves a protonated-amino- or carboxylic-acid-residue as the proton donor. *N*-acetylation that effectively suppresses aggregation could adversely affect the reactivity of the catalyst molecule. Additionally, we obtained somewhat higher activity (43% against natural MP-11) in the previous report [21]. From the three-dimensional model (figure 1), one can find that the haem plane without an axial ligand is open towards the outside and the guest molecule can easily approach the active site to be involved in the catalytic reaction. Accordingly, we can expect that the catalytic activity depends on the primary structure in the vicinity of haem. As the thiol-ene click reaction does not have any preference in reacting with the vinyl group, either 2- or 4-position of haem, the final compound may consist of several structural isomers. Previously, Gray *et al.* examined a low-spin ferric cyanide derivative of MP-8 by using ^1^H and ^13^C NMR and reasonably explained the structure including the thioether bonds [28]. The primary structure of **NAcMP** must be examined similarly in the future for the detailed discussion of specific activity.

As seen in figure 3, the peroxidase activity of **NAcMPm** was 2.7% of natural MP-11. UV–visible and circular dichroic spectral measurements revealed that **NAcMPm** preserved the higher-order structure characteristics of natural MP-11 (electronic supplementary material, figures S6 and S7). On the one hand, class I cytochromes *c* (His/Met coordination) exert peroxidase activity when the haem-Fe loses Met80 coordination [29]. In the ‘push–pull’ mechanism, the histidine-ligated structure has been considered to play the key role in peroxidase reactions [27]. The activity of **NAcMPm** was found to be considerably low comparing with that of **NAcMP**, which could endorse the proposed theory. On the other hand, class I cytochromes *c* show more positive redox potential than class III cytochromes *c* (His/His coordination) [30]. The initial oxidation by H_2_O_2_ proceeds more promptly as the redox potential of the haemperoxidase becomes more negative. Therefore, by examining the redox potential, one may find the cause of low peroxidase activity of **NAcMPm**.

The peroxidase reaction is described by a three-step mechanism involving the formation of compound (cpd) I and II; they are assigned to the iron(IV)-oxo porphyrin π-cation radical and the iron(IV)-oxo species. The overall reaction, including the ferric resting state of the enzyme (E) and the reducing agent, i.e. TMBZ (AH_2_), is given below:

3.1 $$\begin{array}{rl} \text{E}+{\text{H}}_{2}{\text{O}}_{2} & \to \text{cpd}\text{ I}+{\text{H}}_{2}\text{O : }k_{1}, \end{array}$$

3.2 $$\begin{array}{rl} \text{cpd }\text{I}+\text{A}{\text{H}}_{2} & \to \text{cpd}\text{ II}+\text{AH}∙:k_{2} \end{array}$$

3.3 $$\begin{array}{rl} \;\text{and}\;\text{cpd }\text{II}+\text{A}{\text{H}}_{2} & \to \text{ E}+\text{AH}∙+{\text{H}}_{2}\text{O}:k_{3}. \end{array}$$

The rate of reaction (*v*) is given by the steady-state kinetics described by Dunford as follows [31]:

3.4 $$\frac{v}{{\text{[E]}}_{0}}=\frac{2}{\left(1/k_{1}[{\text{H}}_{2}{\text{O}}_{2}])+(1/k_{3}[\text{A}{\text{H}}_{2}]\right)}.$$

AH_2_ exists at a higher concentration that leads to the assumption $k_{3}[\text{A}{\text{H}}_{2}]\gg k_{1}[{\text{H}}_{2}{\text{O}}_{2}],$ and therefore equation (3.4) is further simplified to a simple second-order expression:

3.5 $$v=2k_{1}[{\text{H}}_{2}{\text{O}}_{2}]{\left[\text{E}\right]}_{0}.$$

When treating data in this way (electronic supplementary material, figure S8), simple regression analysis determined the rate constants *k*_1_ and *k*_3_ to be 2.4 × 10^3^ M^−1^ s^−1^ and 1.1 × 10^3^ M^−1^ s^−1^, respectively (*r* = 0.984). The specific rate constant of cpd I formation of **NAcMP** remained low compared with that of *Coprinus cinereus* peroxidase, (6.7 ± 0.2) × 10^6^ M^−1^ s^−1^ [32]. Importantly though, it was of the same order as that reported for natural MP-8, 4778 ± 87 M^−1^ s^−1^ [33].

### 3.3. Spectroelectrochemical study for redox reaction of **NAcMP**

In the following sections, the electrochemical property of **NAcMP** is compared with that of natural MP-11. Here, by using a SOWG device, **NAcMP** was subjected to spectroelectrochemical measurements to probe the chemical species involved.

**Figure 4.**
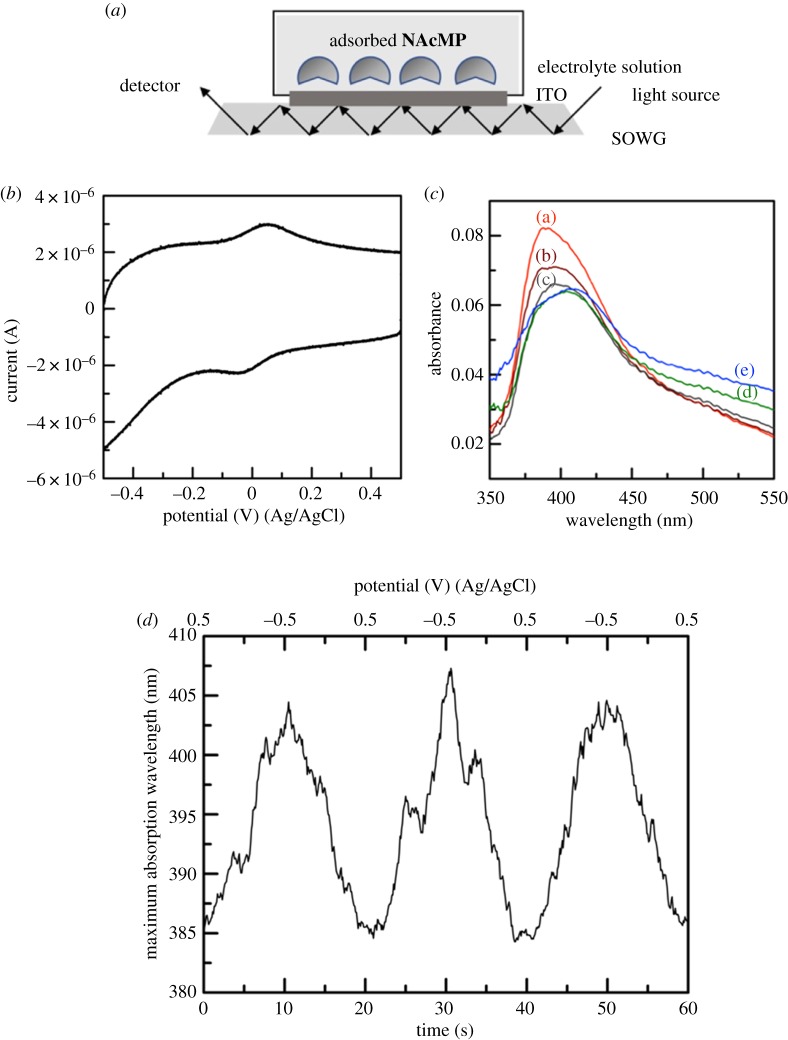
Schematic illustration of spectroelectrochemical measurements using a SOWG device (*a*), and a representative CV for the **NAcMP**–ITO in 1.0 M phosphate buffer (pH 4.2) at 100 mV s^−1^ and 22 ± 2°C (*b*). The UV–visible spectra of **NAcMP**–ITO (*c*) were obtained at various potentials at +500 mV (a), +250 mV (b), 0 mV (c), –250 mV (d) and –500 mV (e), and the time course of *λ*_max_ with repeated potential scanning (*d*).

First, CV measurements confirmed that **NAcMP** undergoes DET reaction at ITO surfaces as is known for natural MP-11 (figure 4*b*). Figure 4*c* summarizes the spectral changes for the **NAcMP**-attached ITO at different potentials. At the initial potential (+500 mV), the **NAcMP**-attached ITO shows an intense UV–visible absorption with a maximum wavelength (*λ*_max_) of 385 nm. The peak moves to a longer wavelength, whereas it monotonically decreases the absorbance as the electrode potential becomes reducible. At −500 mV, *λ*_max_ reached its longest wavelength of 405 nm. **NAcMP** dissolved in phosphate buffer (pH 7.0) showed a Soret band at 408 nm, which moved to a longer wavelength (416 nm) upon reduction by dithionite [21]. The spectroelectrochemical behaviour can be consistent with those obtained in homogeneous solution, considering the short-wavelength shift of *λ*_max_ that is often seen in an adsorption system. The wavelength shift occurred reversibly and reproducibly between 385 and 405 nm even when the potential sweep was repeated dozens of times (figure 4*d*).

As the basis of the electrocatalytic reaction to be studied next, the DET reaction was further examined by conventional CV measurements. We modified Au-disc electrode surfaces with HXT self-assembled monolayers to conveniently accommodate **NAcMP** through hydrophobic interaction (electronic supplementary material, figure S9). Analysis of the CV data determined the electrochemical parameters as summarized in table 1; they were almost consistent with those previously reported [35,36]. Comparing with the SOWG data, one may notice that the formal potential *E*°′, taken as (*E*_pc_ + *E*_pa_)/2, shifts by 340 mV to the negative direction, which could be primarily explained by the pH dependence of *E*°′ (59 mV/pH) [37]. It is also shown that the reduction potential of haem *c* confined in cytochromes *c* can be modified by roughly 500 mV through variations in the degree of haem exposure to solvent [38]. At the HXT-monolayer surface, MP-11 and **NAcMP** showed a considerably negative formal potential, which is presumably solvent-exposed to a great extent. Contrastingly, we can expect that the ITO-adsorbed state effectively hinders the solvent access to the haem as cytochromes *c* attain.

**Table 1.** Electrochemical parameters for the haemprotein–HXT–Au electrode in 0.1 M phosphate buffer solution (pH 7.0) at 25°C.

entry	**NAcMP**	MP-11 (Cyt *c*)
*Γ*/pmol cm^–2^ ^a^	20	23
*E*°′/mV (Ag/AgCl)	–335	–327
Δ*E*_p_/mV	69	123
*k*_DET_/s^–1^ ^b^	1.7	0.71

^a^The value was determined by integration of the charge under the cathodic peak of CV.

^b^The rate constant was calculated by the literature method [34].

### 3.4. Electrocatalytic reaction involving H_2_O_2_

**Figure 5.**
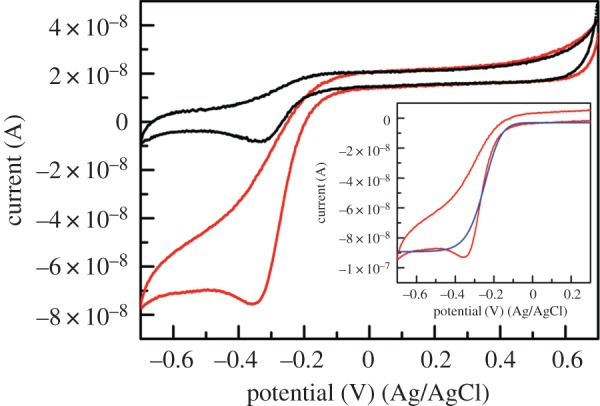
Representative CVs for a **NAcMP**–HXT–Au electrode in 0.1 M phosphate buffer (pH 7) (black lines) or in the presence of 0.33 µM H_2_O_2_ (red lines). Scan rate 10 mV s^−1^, temperature 22 ± 2°C. The inset compares the background-subtracted experimental CV (red lines) and the theoretical current–voltage curve during the forward scan (blue line). Owing to a quasi-reversible direct electron transfer reaction, replication of the reversed-scan wave was unsatisfactory.

As shown in figure 5, the **NAcMP**–Au electrode accepts H_2_O_2_ as an oxidizing agent to give characteristic CV responses. The initially observed, peak-shaped cathodic current (equation (3.6)) increased to a considerable extent and changed to a sigmoidal curve, which is typical of a steady-state reaction. Iron(II) is oxidized to a ferryl group by H_2_O_2_ in several reactions [39]. Therefore, as the primary component, the one-electron-reduced product of haemin can account for the behaviour (equation (3.7)):

3.6 $$\begin{array}{rl} & \text{NAcMP}\text{-Fe}(\text{III})+{\text{e}}^{-}⇌\text{NAcMP}\text{-Fe}(\text{II}):k_{\mathrm{DET}}, \end{array}$$

3.7 $$\begin{array}{rl} & \text{NAcMP}\text{-Fe}(\text{II})+{\text{H}}_{2}{\text{O}}_{2}⇌\text{NAcMP}\text{-Fe}(\text{IV})=\text{O}+{\text{H}}_{2}\text{O} \end{array}$$

3.8 $$\begin{array}{rl} \;\text{and}\; & \text{NAcMP}\text{-Fe}(\text{IV})=\text{O}+{\text{e}}^{-}+2{\text{H}}^{+}⇌\text{NAcMP}\text{-Fe}(\text{III})+{\text{H}}_{2}\text{O}. \end{array}$$

Because the haem ferryl group has a far more positive *E*° value than the ferric haem, it is immediately reduced at the electrode potential (equation (3.8)). The regenerated substrates **NAcMP**-Fe(III) are again involved in equation (3.6) to achieve a steady state.

We simply used digital simulations for characterizing the substrate-recycling DET reaction adopting an assumption. The redox-active species exist only at about 10^–12^ mol cm^–2^ level on the electrode surface, which is extremely small when compared with H_2_O_2_ dissolved in the solution phase. Given this, the second-order interfacial reaction was simplified to a pseudo-first-order reaction with the rate constant *k*_ex_ (equation (3.7′)):

3.7′ $$\text{NAcMP}\text{-Fe}(\text{II})\to \text{NAcMP}\text{-Fe}(\text{IV})=\text{O}:k_{\mathrm{ex}}.$$

The parameter sets that were determined from the best-fit data are summarized in table 2. It is apparent that *E*°′ of **NAcMP** shifted to the positive direction by 135 mV, indicating that catalytic action was occurring. It is reported that coordination of the haem with certain types of the sixth-ligands including *N*-acetylmethionine (AcMet) caused a positive shift of the *E*°′ of the haem. An *E*°′ is the expression of the relative stability of the two redox partners under the particular conditions including the medium used for measurements. Currently, we are not aware whether the H_2_O_2_ coordination occurs preferentially to the haem-Fe(II) or the haem-Fe(III) in **NAcMP**. Yet, we expect that, similarly with AcMet, the affinity of H_2_O_2_ coordination with haem-Fe(II) is considerably higher than that of the oxidized peptide, which increased the haem reduction potential. The DET rate constant was classified as moderate reaction similar to the noncatalytic system. The rate constant for substrate regeneration, 1.8 s^−1^, represents a rather slow turnover frequency. In table 2 are given the electrocatalytic parameters obtained for natural MP-11 (electronic supplementary material, figure S10). In previous reports, Willner *et al*. examined the maximum current at H_2_O_2_ saturation that corresponds to *v*_max_ in the Michaelis–Menten model; the MP-11-modified electrode was determined to be −1.0 µA [40]. Later, a bis-histidine-ligated unfolded cytochrome *c* mutant was examined in a similar way by Scheller *et al.*; it was found to be 5.18 µA cm^−2^ [41]. As can be seen from the unit of current density, if the Faraday and the electroactive surface concentration divide the data, it becomes the pseudo-first-order rate constant. Comparison of the data concluded that the rate constant for both **NAcMP** and natural MP-11 was one order larger than that of the previous report, and was of the same order as the unfolded cytochrome *c* mutant.

**Table 2.** Global parameters estimated by theoretical simulation of the background-subtracted experimental current–voltage profiles.

entry	**NAcMP**	MP-11 (Cyt *c*)
*Γ*/pmol cm^–2^ ^a^	20	23
*E*°′_app_/mV (Ag/AgCl)	–200	–230
*k*_DET_/s^–1^	0.70	0.60
*α* ^b^	0.5	0.5
*C*_dl_/µF ^a^	0.3	0.5
*k*_ex_/s^–1^	1.8	1.6

^a^These values were determined from CVs obtained in 0.1 M phosphate buffer solution without H_2_O_2_.

^b^The parameter was fixed at 0.5 by assuming a symmetrical energy barrier of the electrode reaction.

## 4. Conclusion

Here, we reported the first totally synthetic MP-11. By taking advantage of the thiol-ene click reaction, we have successfully reconstituted haem *c* into the host peptide, which one could not achieve so far. We have also succeeded to visualize, with animated AFM images, that the spatial structure of protein remarkably changes before and after the treatment. Oxidation of TMBZ by hydrogen peroxide confirmed that the synthetic haemperoxidase displayed a similar degree of enzymatic activity to that of natural MP-11. Additionally, the synthetic material achieved an electrochemical catalytic system that will be useful for H_2_O_2_-sensing platform. Computer simulation by trial and error well reproduced the catalytic current–voltage curve and simultaneously, estimated the catalytic rate constant that seemed appropriate. In all experiments, the results of the synthetic MP-11 were almost identical to those of its naturally occurring counterpart. Therefore, we concluded that our approach is a convenient means of MP-11 synthesis that has no reliance on nature. From the viewpoint of improved efficiency of enzymatic reaction, a synthetic mutant, **NAcMPm**, was disappointing. However, **NAcMPm** seems an example of proof that our approach allows us various peptides in hand. We expect that our totally synthetic strategy will be a useful means in preparing MPs regardless of natural or nonnatural sequence.

## Supplementary Material

nakanokoji-SI-R1

## Supplementary Material

nakanokoji-SI-AFM

## Data accessibility

The electronic supplementary material, including the synthetic procedures, analysis data, UV–visible spectra, CD spectra and cyclic voltammograms, is available to support the paper. Additionally, the animated versions of AFM images for both the undecapeptide and the synthetic MP-11 are accessible as a PowerPoint slideshow.
