# Supplementary material for: Totally synthetic microperoxidase-11
Source: R Soc Open Sci. 2018 May 23;5(5):172311. doi: 10.1098/rsos.172311 (PMC5990835; doi:10.1098/rsos.172311)
Supplement: nakanokoji-SI-R1 [file rsos172311supp1.pdf]

Supporting Information

Totally Synthetic Microperoxidase-11

Junichi Tanabe, Koji Nakano, Ryutaro Hirata, Toshiki Himeno, Ryoich Ishimatsu, Toshihiko Imato, Hirotake Okabe, and Naoki Matsuda

Contents

1. *Synthetic procedure and analysis data* 2
   1.1 Chemicals 2
   1.2 Synthesis of undecapeptide 2
   1.3 Reconstitution of undecapeptide by thiol-ene click reaction 5
   1.4 Synthesis of undecapeptide mutant 7
   1.5 Reconstitution of undecapeptide mutant by thiol-ene click reaction 10
2. *AFM imaging in water* 12
3. *UV-vis spectrum of MP-11 mutant* 13
4. *CD spectrum of MP-11 mutant* 14
5. *Peroxidase-activity measurements for* ***NAcMP*** *and steady-state analysis* 15
6. *Electrochemical and electrocatalytic measurements* 17
7. *References* 19

1. *Synthetic procedure and analysis data*

1.1 Chemicals

Rink Amide AM resin (200-400 mesh, 0.62 mmol g^{-1} loading) was purchased from Merck Millipore (Darmstadt, Germany). Fmoc-protected amino acids (Fmoc-AA-OH) were obtained from Watanabe Chemical Industries, Ltd (Hiroshima, Japan). Similarly, piperidine, o-(1H-benzotriazol-1-yl)-*N,N,N',N'*-tetramethyluronium hexafluorophosphate (HBTU), *N*-hydroxybenzotriazole (HOBt), and diisopropylethylamine (DIPEA) were supplied from the company. HPLC grade dimethyl formamide (DMF), dichloromethane (DCM), trifluoroacetic acid (TFA), acetic anhydride (Ac_2O), triisopropylsilane (TIS), and 1,2-ethanedithiol (EDT) were bought from Wako Pure Chemical Industries Ltd (Osaka, Japan). Commercially available microperoxidase 11 sodium salt (Sigma-Aldrich Co. LLC, St. Louis, MO) was used for comparison measurements. Other chemicals were reagent grade and used as received.

1.2 Synthesis of undecapeptide

The undecapeptide, VQKCAQCHTVE, were prepared by solid phase peptide synthesis (SPPS) at a 0.1-mmol scale in a manually operated microwave synthesis system (Discover® SP, CEM Co, Matthews, NC, USA). The corresponding amount of Rink Amide AM resin (161 mg) was settled in a reaction vessel and was swollen in 7 mL DMF for overnight prior to use. For fmoc deprotection, 0.1 M HOBt in 20%-piperidine/DMF solution was used. The activator base solution was 5%-DIPEA in DMF. The cleavage cocktail was prepared by mixing TFA (4.7 mL), TIS (0.05 mL), EDT (0.125 mL), and deionized water (0.125 mL). The synthetic procedure was as given below.

1. Wash the resin with 7 mL DMF and drain the solvent by filtration under reduced pressure. This treatment was repeated three times.
2. Fmoc deprotection of solid support. Add the fmoc-deprotection solution (7 ml). Apply microwave (MW) at 20 W The reaction temperature was set 75 °C and the reaction mixture was left to react for 3 min and then, drain the solution. Wash the resin with 7 mL DMF, swirl for 10 s, and drain the solvent. Repeat four times.
3. AA coupling with resin. Each 0.5-mmol amount of Fmoc-Glu(OtBu)-OH and

HBTU were dissolved into a 4-mL portion of the activator base solution and the solution was added to the resin. Apply MW (20 W, 75 °C, 5 min) and then, drain the solvent. Wash the resin with 7 mL DMF, swirl for 10 s, and drain the solvent. Repeat four times. As occasion arose, Kaiser test confirmed that the reaction satisfactory proceed.

4. Peptide chain elongation. Each 0.5-mmol amount of Fmoc-AA-OH and HBTU were dissolved into a 4-mL portion of the activator base solution. Repeat step 2 and 3 for each successive amino acid in the sequence in the following order: Fmoc-Val-OH, Fmoc-Thr(tBu)-OH, Fmoc-His(Trt)-OH, Fmoc-Cys(Trt)-OH, Fmoc-Gln(Trt)-OH, Fmoc-Ala-OH, Fmoc-Cys(Trt)-OH, Fmoc-Lys(boc)-OH, Fmoc-Gln(Trt)-OH, and Fmoc-Val-OH. Kaiser test confirmed the final coupling reaction. Wash the resin with 7 mL DMF, swirl for 10 s, and drain the solvent. Repeat four times.
5. N-terminal acetyl capping. Add the fmoc-deprotection solution (7 ml). Apply microwave (MW, 20 W, 75 °C, 3 min) and drain the solvent. Wash the resin with 7 mL DMF, swirl for 10 s, and drain the solvent. This procedure was repeated four times. Subsequently, wash the resin with 7 mL DCM, swirl for 10 s, and drain the solvent. This procedure was repeated four times. Add an 8-mL portion of 25% Ac_2O in DMF, stand for 5 min, and drain the solvent. Wash with 7 mL DCM, swirl for 10 s, and drain the solvent. Repeat four times.
6. Peptide release and side chain deprotection. Add the cleavage cocktail, swirl for 10 s, and apply MW (20 W, 38°C, 30 min). Transfer the solution cleaved peptide by filtration under reduced pressure. Prepare 34-pieces of clean 15-mL centrifuge tubes and into each tube placed was a 10-mL portion of cold diethyl ether. The equally-divided portions of the peptide solution were poured into the tubes and allow the peptide to precipitate for at least 30 min. Centrifuge the precipitated peptide for four-successive times (120,000 rpm, 30 min) at 0 °C to isolate the crude product and then, carefully decantate the supernatant. Repeat four times. The crude peptide thus obtained was dried under reduced pressure overnight.
7. Precipitation separation. Dissolve the peptide in 15 mL deionized water. Freeze the sample with liquid nitrogen and lyophilized overnight. The crude product was obtained as white precipitate in glacial ether: Yield of the crude product 0.1981 g

(153%).

8. Peptide analysis. The undecapeptide was examined by HPLC and was chromatographically purified on a C18-column (4.6 x 250 mm) if necessary (buffer A: 0.05% TFA in water; buffer B: 0.01% TFA in acetonitrile). HPLC elution (1 mL min^{-1}) was initialized at 85% buffer A that was linearly changed at time to 30% (0–30 min), 20% (30–35 min), 95% (35–45 min), and 95% (45–50 min). MALDI TOFF-MS using α-cyano-4-hydroxycinnamic acid as matrix analysed the obtained products; m/z 1288.324 (M-H^+. $C_{52}H_{86}N_{16}O_{18}S_2$ requires 1287.47).

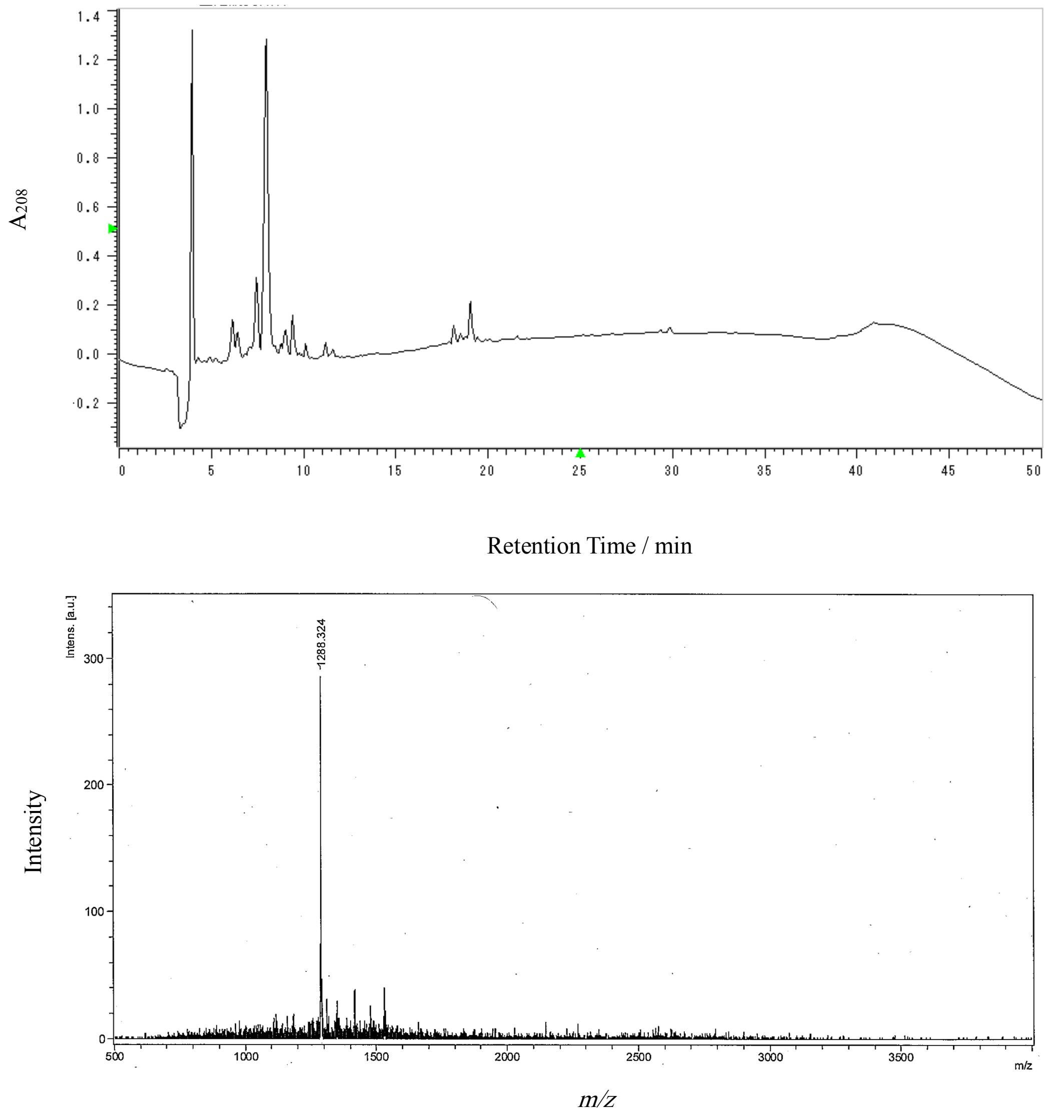

Figure S1. Results of HPLC separation (top) and MALDI-TOF-MS analysis (bottom) for the undecapeptide.

1.3 Reconstitution of undecapeptide by thiol-ene click reaction

For the protein-reconstitution experiments, hemin and 2-hydroxy-1-[4-(2-hydroethoxy)phenyl]-2-methylpropan-1-one (photoinitiator) were purchased from Tokyo Chemical Industry Co., Ltd (Tokyo, Japan). Dithiothreitol (DTT) were supplied from Wako Pure Chemical Industries Ltd (Osaka, Japan). For control measurements, naturally-derived microperoxydase-11 was bought from Sigma-Aldrich Japan Co. LLC (Tokyo, Japan). Other chemicals were reagent grade and used as received.

The undecapaptide was reconstituted with the prosthetic group by using thiol-ene click reaction[1–4]. A weighed amount of the undecapeptide (6.4 mg, 5.0 μmol) was dissolved into 3.5-mL portion of aqueous 50%-THF solution that was, by slowly bubbling nitrogen gas, deoxygenated beforehand. The solution was combined with a 200-μL volume of aqueous 0.1 M NaOH solution containing 4.0-mg of hemin (6.0 μmol) and into the reaction mixture, was added each one-third microspatula full of the photoinitiator, DTT, and sodium dithionite. The reaction was allowed to proceed by exposing to UV light (365 nm, 40 mW cm^{-2}) for 60 min at RT. The undecapeptide in the corresponding holo structure that was identified by comparison experiments using naturally-derived microperoxidase-11, was chromatographically purified on a C18-column. The HPLC conditions were the same as those used for undecapaptide. The fractions that were eluted from 12.5 min to 17.5 min, were combined and were subjected for centrifugal concentration. This resulted trace amount of red-brown powder after lyophilisation: Yield trace, we obtained, as a typical case, 1.0%-of-yield when 5-μmol of apo-**NAcMP** was subjected for the reaction, repeating 4 times, and the combined solution was purified by HPLC. MALDI TOFF-MS using α-cyano-4-hydroxycinnamic acid as matrix analysed the obtained products; *m/z* 1900.721 ($M\text{-}H^+$. $C_{86}H_{118}FeN_{20}O_{22}S_2$ requires 1903.97). A fragmentation peak at *m/z* 1285.364 ($M\text{-}H^+$ for **NAcMP**, $C_{52}H_{87}N_{16}O_{18}S_2$ requires 1287.58), which further dimerized to give a peak at *m/z* 2571.491 (M^+ for $C_{104}H_{173}N_{32}O_{36}S_4$ requires 2574.157). An ionization product formed by elimination of imidazolyl group of His ($M\text{-}H^+$ for $C_{82}H_{115}FeN_{18}O_{22}S_2$ requires 1823.722), which further decomposed in part may give a fragmentation peak at *m/z* 1815.779.

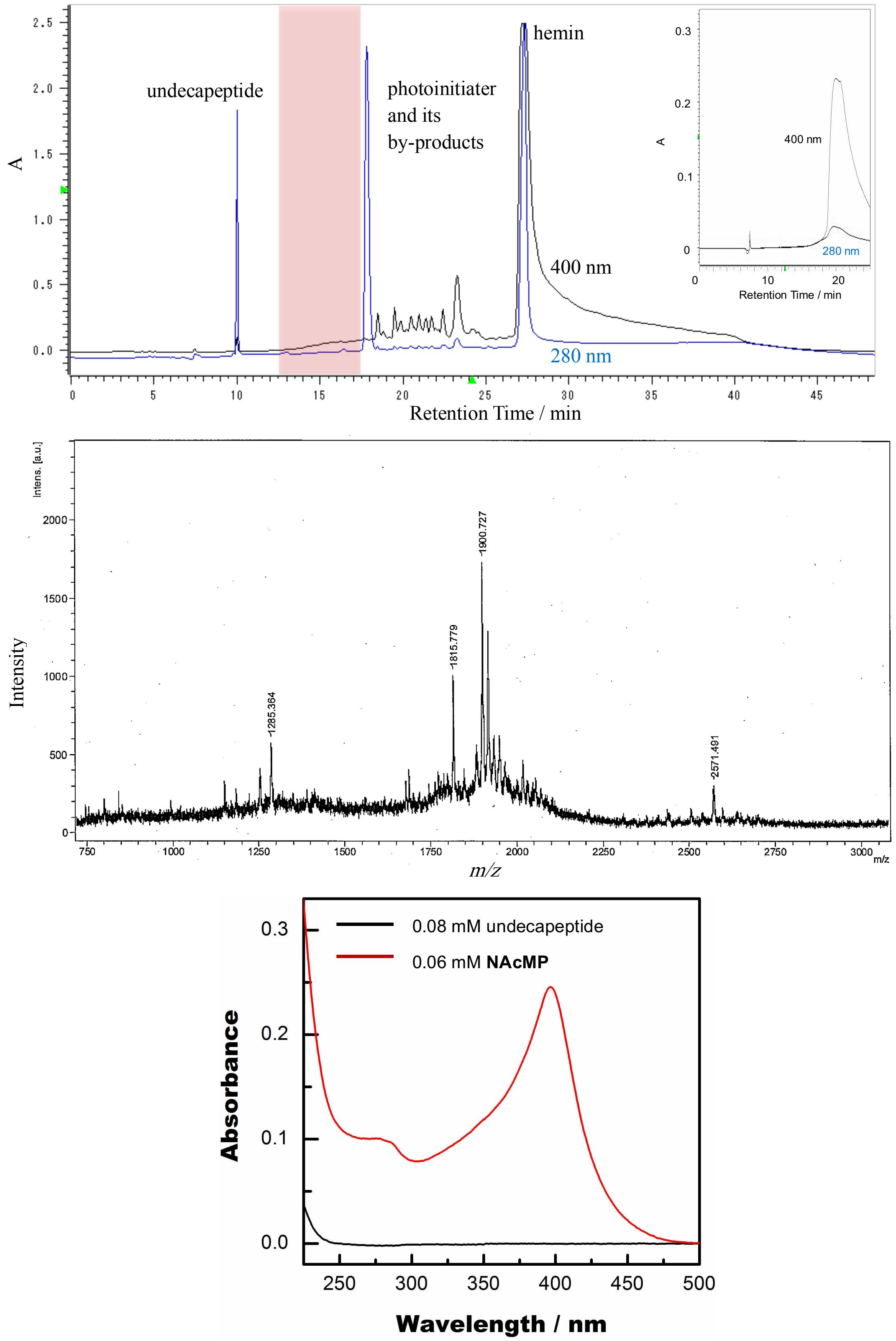

Figure S2. Results of HPLC separation (top), MALDI-TOF-MS analysis (middle), and UV-vis spectra in TFE (bottom, 0.1 cm cells). The inset shows the chromatogram for natural MP-11.

1.4 Synthesis of undecapeptide mutant

The corresponding amount of Rink Amide AM resin (161 mg) was settled in a reaction vessel and was swollen in 7 mL DMF for overnight prior to use. For fmoc deprotection, 0.1 M HOBt in 20%-piperidine/DMF solution was used. The activator base solution was 5%-DIPEA in DMF. The cleavage cocktail was prepared by mixing TFA (4.7 mL), TIS (0.05 mL), EDT (0.125 mL), and deionized water (0.125 mL). The synthetic procedure for the undecapeptide mutant, VQKCAQC***M***TVE (H8M), was as given below.

1. Wash the resin with 7 mL DMF and drain the solvent by filtration under reduced pressure. This treatment was repeated three times.
2. Fmoc deprotection of solid support. Add the fmoc-deprotection solution (7 ml). Apply microwave (MW) at 20 W The reaction temperature was set 75 °C and the reaction mixture was left to react for 3 min and then, drain the solution. Wash the resin with 7 mL DMF, swirl for 10 s, and drain the solvent. Repeat four times.
3. AA coupling with resin. Each 0.5-mmol amount of Fmoc-Glu(OtBu)-OH and HBTU were dissolved into a 4-mL portion of the activator base solution and the solution was added to the resin. Apply MW (20 W, 75 °C, 5 min) and then, drain the solvent. Wash the resin with 7 mL DMF, swirl for 10 s, and drain the solvent. Repeat four times. As occasion arose, Kaiser test confirmed that the reaction satisfactory proceed.
4. Peptide chain elongation. Each 0.5-mmol amount of Fmoc-AA-OH and HBTU were dissolved into a 4-mL portion of the activator base solution. Repeat step 2 and 3 for each successive amino acid in the sequence in the following order: Fmoc-Val-OH, Fmoc-Thr(tBu)-OH, Fmoc-Met-OH, Fmoc-Cys(Trt)-OH, Fmoc-Gln(Trt)-OH, Fmoc-Ala-OH, Fmoc-Cys(Trt)-OH, Fmoc-Lys(boc)-OH, Fmoc-Gln(Trt)-OH, and Fmoc-Val-OH. Kaiser test confirmed the final coupling reaction. Wash the resin with 7 mL DMF, swirl for 10 s, and drain the solvent. Repeat four times.
5. N-terminal acetyl capping. Add the fmoc-deprotection solution (7 ml). Apply microwave (MW, 20 W, 75 °C, 3 min) and drain the solvent. Wash the resin with 7 mL DMF, swirl for 10 s, and drain the solvent. This procedure was repeated

four times. Subsequently, wash the resin with 7 mL DCM, swirl for 10 s, and drain the solvent. This procedure was repeated four times. Add an 8-mL portion of 25% Ac_2O in DMF, stand for 5 min, and drain the solvent. Wash with 7 mL DCM, swirl for 10 s, and drain the solvent. Repeat four times.

6. Peptide release and side chain deprotection. Add the cleavage cocktail, swirl for 10 s, and apply MW (20 W, 38°C, 30 min). Transfer the solution cleaved peptide by filtration under reduced pressure. Prepare 34-pieces of clean 15-mL centrifuge tubes and into each tube placed was a 10-mL portion of cold diethyl ether. The equally-divided portions of the peptide solution were poured into the tubes and allow the peptide to precipitate for at least 30 min. Centrifuge the precipitated peptide for four-successive times (120,000 rpm, 30 min) at 0 °C to isolate the crude product and then, carefully decantate the supernatant. Repeat four times. The crude peptide thus obtained was dried under reduced pressure overnight.
7. Precipitation separation. Dissolve the peptide in 15 mL deionized water. Freeze the sample with liquid nitrogen and lyophilized overnight. The crude product was obtained as white precipitate in glacial ether: Yield of the crude product 0.1388 g (108%).
8. Peptide analysis. The undecapeptide was examined by HPLC and was chromatographically purified on a C18-column (4.6 x 250 mm) if necessary (buffer A: 0.05% TFA in water; buffer B: 0.01% TFA in acetonitrile). HPLC elution (1 mL min^{-1}) was initialized at 85% buffer A that was linearly changed at time to 30% (0–30 min), 20% (30–35 min), 95% (35–45 min), and 95% (45–50 min). MALDI TOFF-MS using α-cyano-4-hydroxycinnamic acid as matrix analysed the obtained products; m/z 1281.974 ($M-H^+$. $C_{51}H_{89}N_{14}O_{18}S_3$ requires 1281.564).

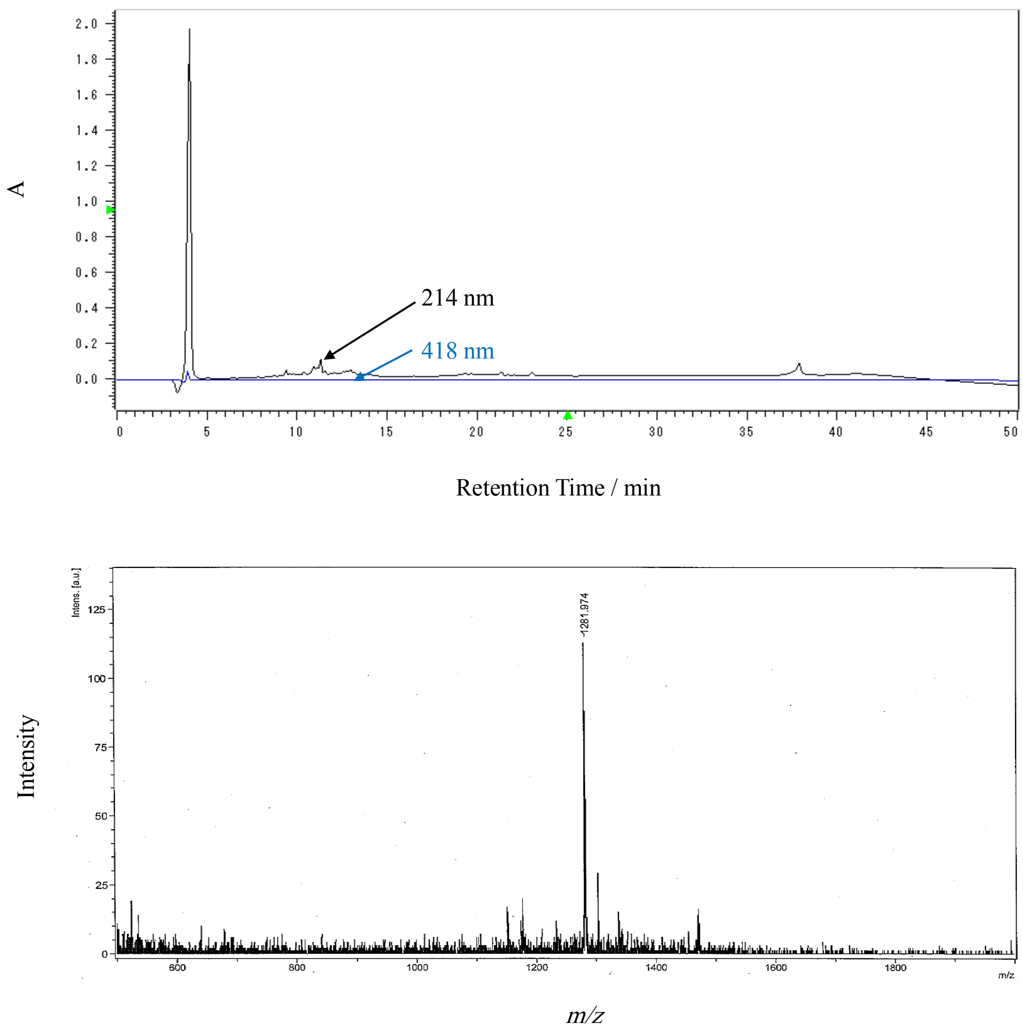

Figure S3. Results of HPLC separation (top) and MALDI-TOF-MS analysis (bottom) for the undecapeptide mutant.

1.5 Reconstitution of undecapeptide mutant by thiol-ene click reaction

The undecapaptide was reconstituted with the prosthetic group by using thiol-ene click reaction[1–4]. A weighed amount of the undecapeptide (6.4 mg, 5.0 μmol) was dissolved into 3.5-mL portion of aqueous 50%-THF solution that was, by slowly bubbling nitrogen gas, deoxygenated beforehand. The solution was combined with a 200-μL volume of aqueous 0.1 M NaOH solution containing 4.0-mg of hemin (6.0 μmol) and into the reaction mixture, was added each one-third microspatula full of the photoinitiator, DTT, and sodium dithionite. The reaction was allowed to proceed by exposing to UV light (365 nm, 40 mW cm^{-2}) for 60 min at RT. The undecapeptide in the corresponding holo structure that was identified by comparison experiments using naturally-derived microperoxidase-11, was chromatographically purified on a C18-column. The HPLC conditions were the same as those used for undecapaptide. The fractions that were eluted from 13.3 min to 17.1 min, were combined and were subjected for centrifugal concentration. This resulted trace amount of red-brown powder after lyophilisation: Yield trace. MALDI TOFF-MS using α-cyano-4-hydroxycinnamic acid as matrix analysed the obtained products; *m/z* 1898.557 ($M-H^+$. $C_{85}H_{120}FeN_{18}O_{22}S_3$ requires 1897.741). A fragmentation peak at *m/z* 1303.360 ($M-Na^+$ for apo MP-11 variant, $C_{51}H_{88}N_{14}NaO_{18}S_3$ requires 1303.546), which further bound to apo MP-11 variant to give a peak at *m/z* 2585.108 ($M-Na^+$ for $C_{102}H_{176}N_{28}NaO_{36}S_6$ requires 2584.102).

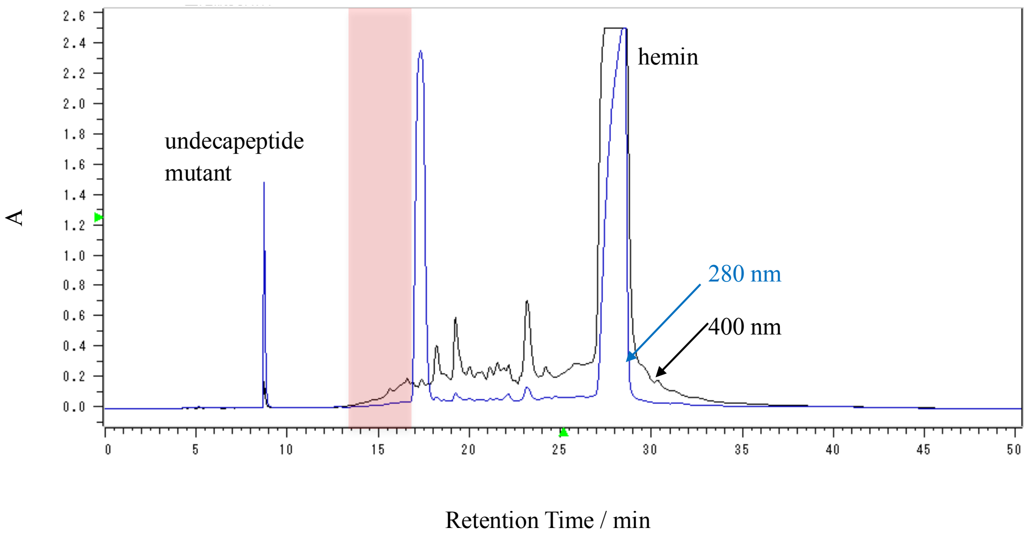

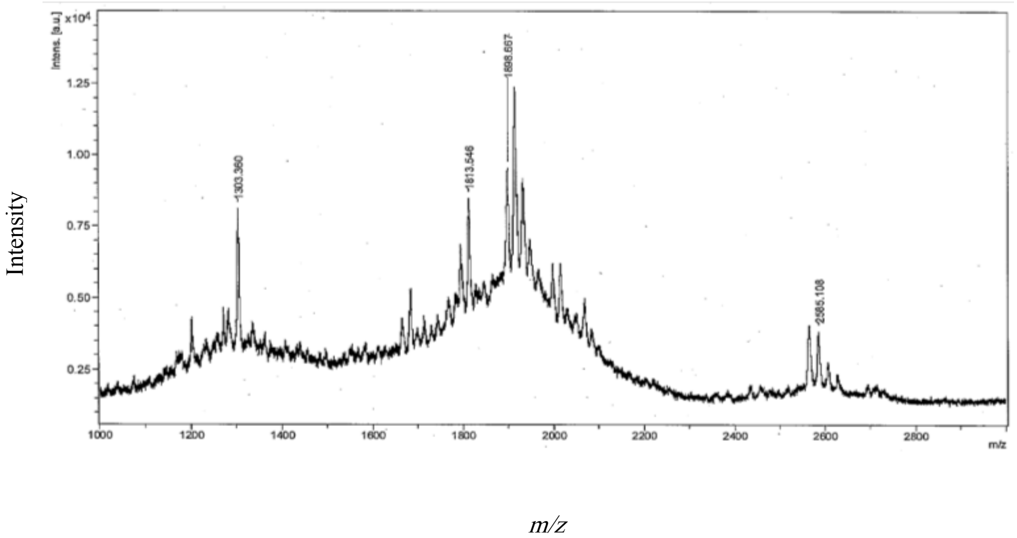

Figure S4. Results of HPLC separation (top) and MALDI-TOF-MS analysis (bottom) for the undecapeptide mutant after reconstitution of hemin.

2. AFM imaging in water

(A)

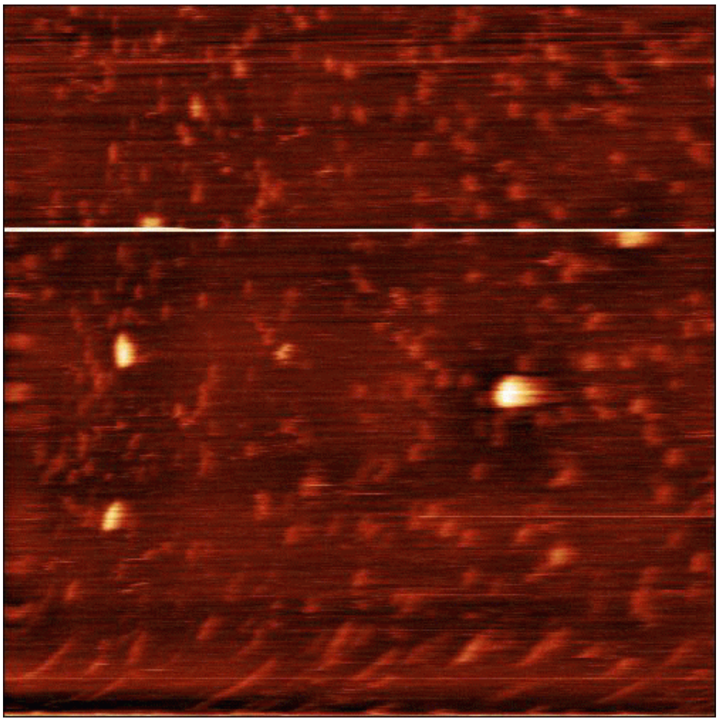

(B)

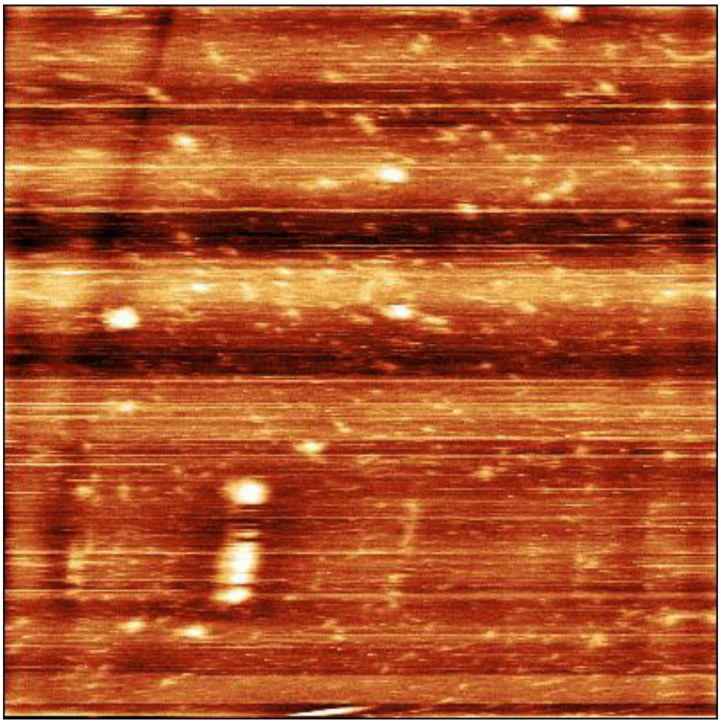

Figure S5. Animated versions of representative AFM images (500 x 500 nm) for the undecapeptide **(A)** and **NAcMP (B)** in H_2O. Animations are available in slideshow of the PowerPoint file attached in ESI.

3. UV-vis spectrum of MP-11 mutant

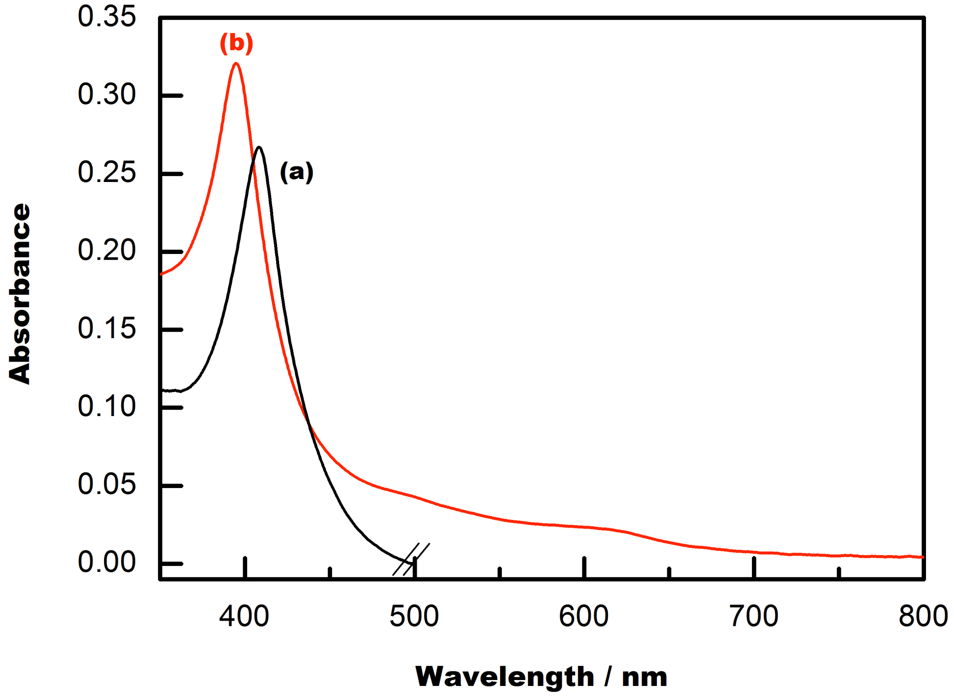

Figure S6. UV-vis spectrum for 2x10^{-5} M **NAcMPm** in 0.01 M phosphate buffer (pH 7) **(b)**. The path-length of the cell was 0.2 cm and temperature was 25 °C. The spectral data for 2x10^{-5} M **NAcMP (a)** was reproduced from Ref. 5.

4. CD spectrum of MP-11 mutant

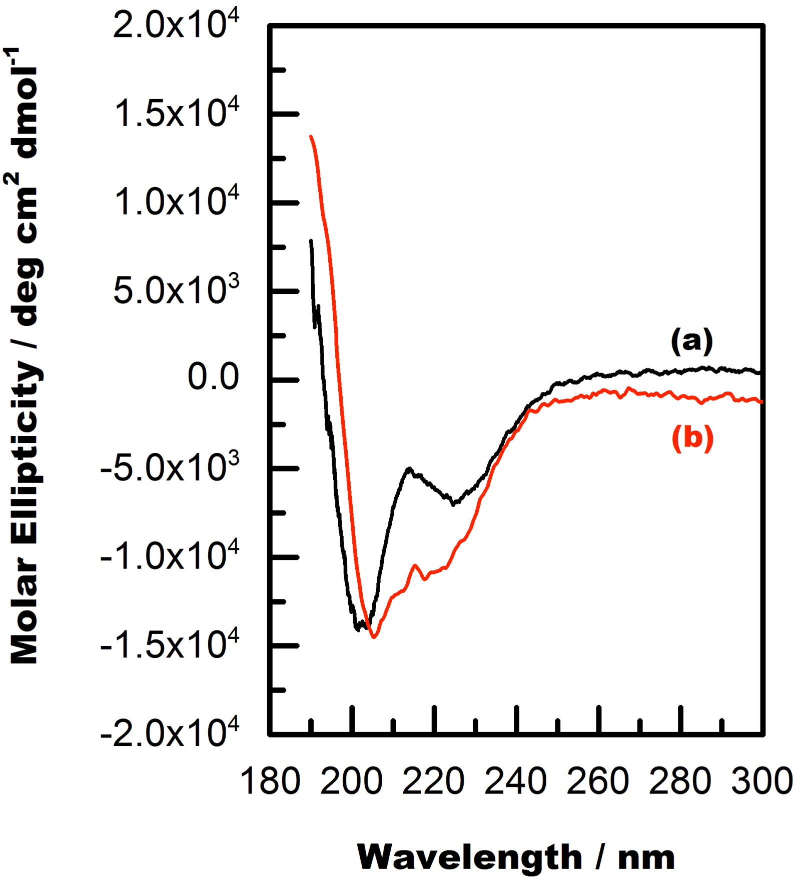

Figure S7. CD spectrum for 53 μM **NAcMPm** dissolved in TFE **(b)**. The path-length of the cell was 1 cm and temperature was 25 °C. The spectral data for 64 μM **NAcMP (a)** was reproduced from Ref. 5.

5. Peroxidase-activity measurements for NAcMP and steady-state analysis

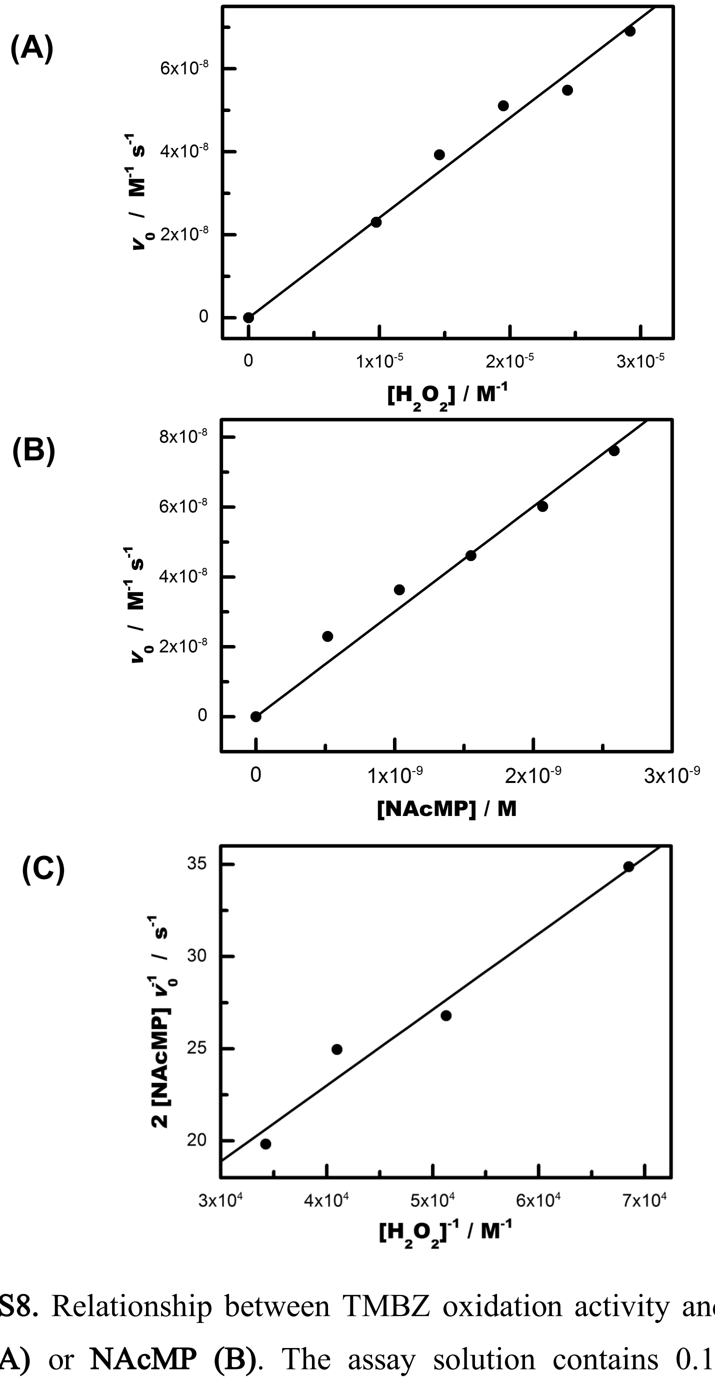

Figure S8. Relationship between TMBZ oxidation activity and concentration either of H_2O_2 **(A)** or **NAcMP (B)**. The assay solution contains 0.14 mM TMBZ (0.01 M phosphate buffer, pH 7) and either of 40 μM **NAcMP** (top) or 15 μM H_2O_2 (bottom) and temperature was 37 °C. In **(C)** given are the plots according to steady-state kinetics (Eq. 4) for **NAcMP** obtained with various H_2O_2 concentrations at 37 °C. The assay solution (0.1 M phosphate buffer) contains 0.68 μM **NAcMP**, 0.14 mM TMBZ, and 15 μM H_2O_2.

6. *Electrochemical and electrocatalytic measurements*

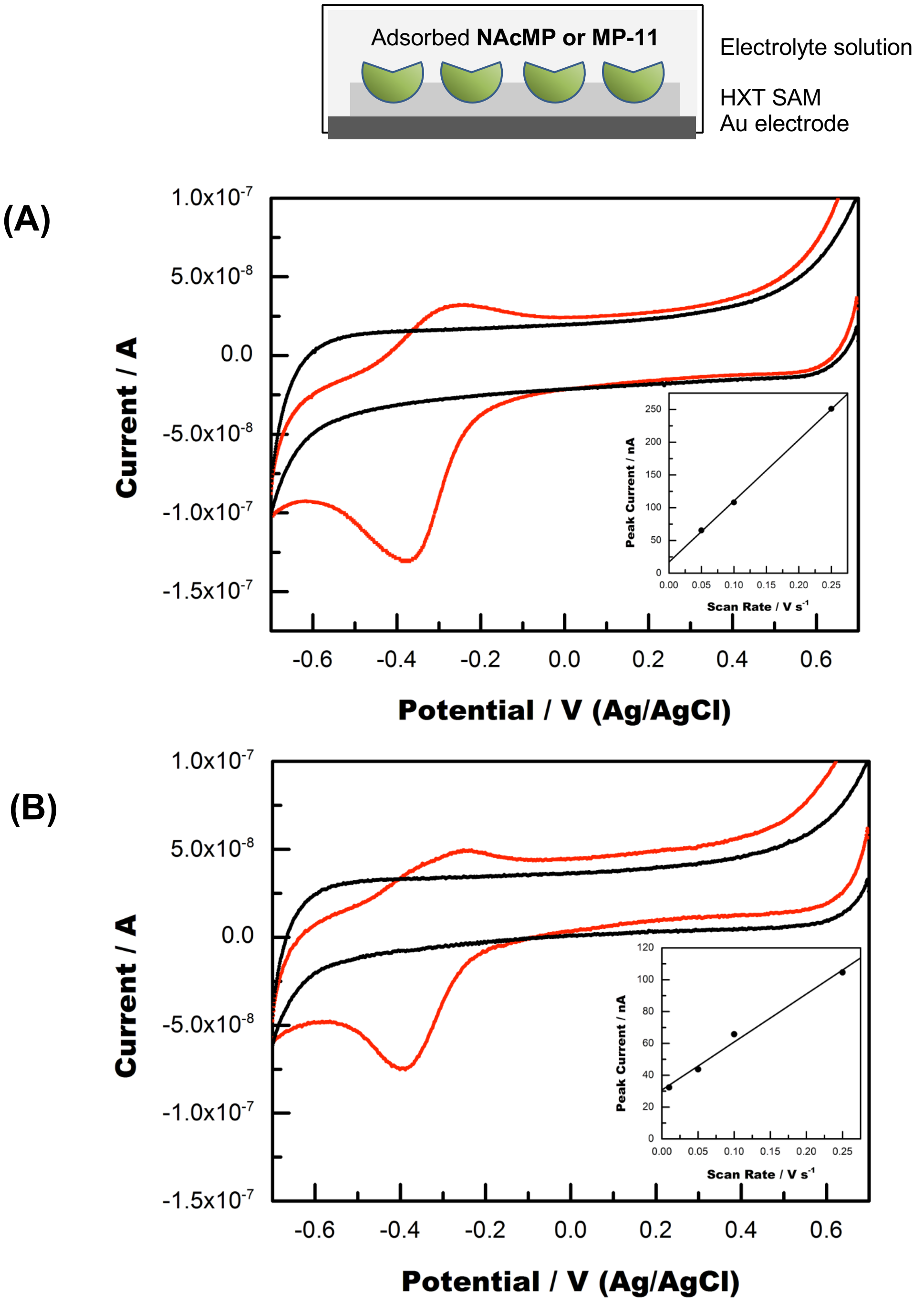

Figure S9. Representative results of CV measurements for **NAcMP**–HXT–Au electrode **(A)** and MP-11(natural)–HXT–Au electrode **(B)** in 0.1 M phosphate buffer (pH 7) at 100 mV s^{-1} and 22±2 °C (red lines). CVs for the HXT-Au electrodes without the corresponding heampeptide were gives as black lines. Each inset shows the scan-rate dependence of the magnitude of reduction peak current.

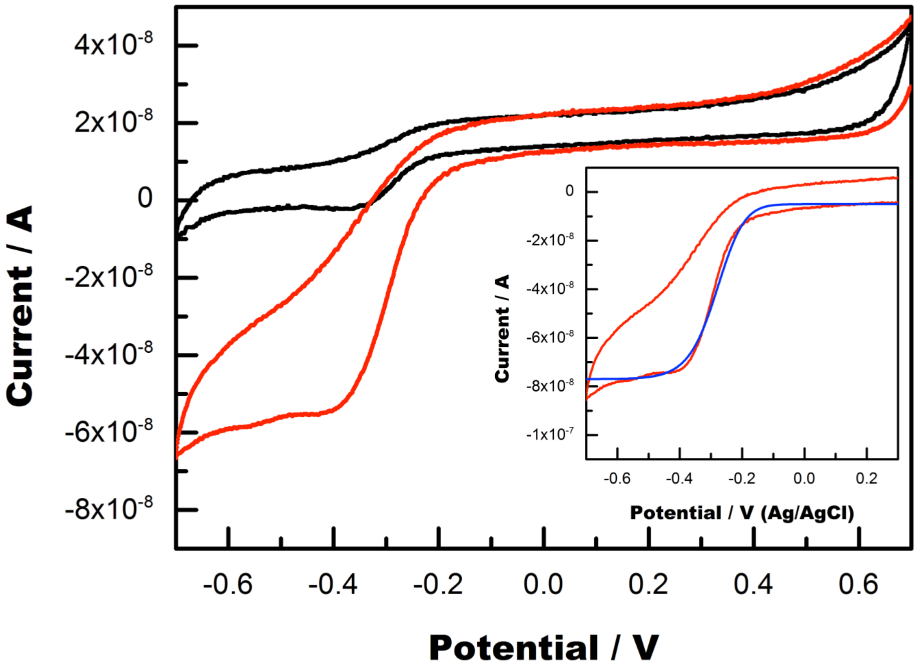

Figure S10. Representative CVs for a MP-11(natural)–HXT–Au electrode either in 0.1 M phosphate buffer (pH 7) (black lines) or in the presence of 0.33 μM H_2O_2 (red lines). Scan rate 10 mV s^{-1}, temperature 22±2 °C. The inset compares the background-subtracted experimental CV (red lines) and the theoretical current–voltage curve during the forward scan (blue lines). Owing to a quasi-reversible direct electron transfer reaction, replication of the reversed-scan wave was unsatisfactory.

7. References

1. Hoppmann, C., Schmieder, P., Heinrich, N., and Beyermann, M. Photoswitchable Click Amino Acids: Light Control of Conformation and Bioactivity, *ChemBioChem*, **12**, 2555–2559 (2011).
2. Hoppmann, C., Kühne, R., and Beyermann, M. Intramolecular bridges formed by photoswitchable click amino acids, *Beilstein J. Org. Chem*, **8**, 884–888 (2012).
3. Ascoli, F., Fanelli, M. R. R., and Antonini, E. Preparation and properties of apohemoglobin and reconstituted hemoglobins, *Meth. Enzymol.*, **76**, 72–87 (1981).
4. Wagner, G. C., Perez, M., Toscano, Jr., W. A., and Gunsalus, I. C. Apoprotein formation and heme reconstitution of cytochrome P-450_{cam}, *J. Biol. Chem.*, **256**, 6262–6265 (1981).
5. Nakano, K., Tanabe, J., Ishimatsu, R., Imato, T. Monolithic peptide-nucleic acid hybrid functioning as an artificial microperoxidase, *Bioconjugate Chem.,* **28**, 2031–2034 (2017).
