# Supplementary figures and images for: Totally synthetic microperoxidase-11
Source: R Soc Open Sci. 2018 May 23;5(5):172311. doi: 10.1098/rsos.172311 (PMC5990835; doi:10.1098/rsos.172311)

## Slide 1
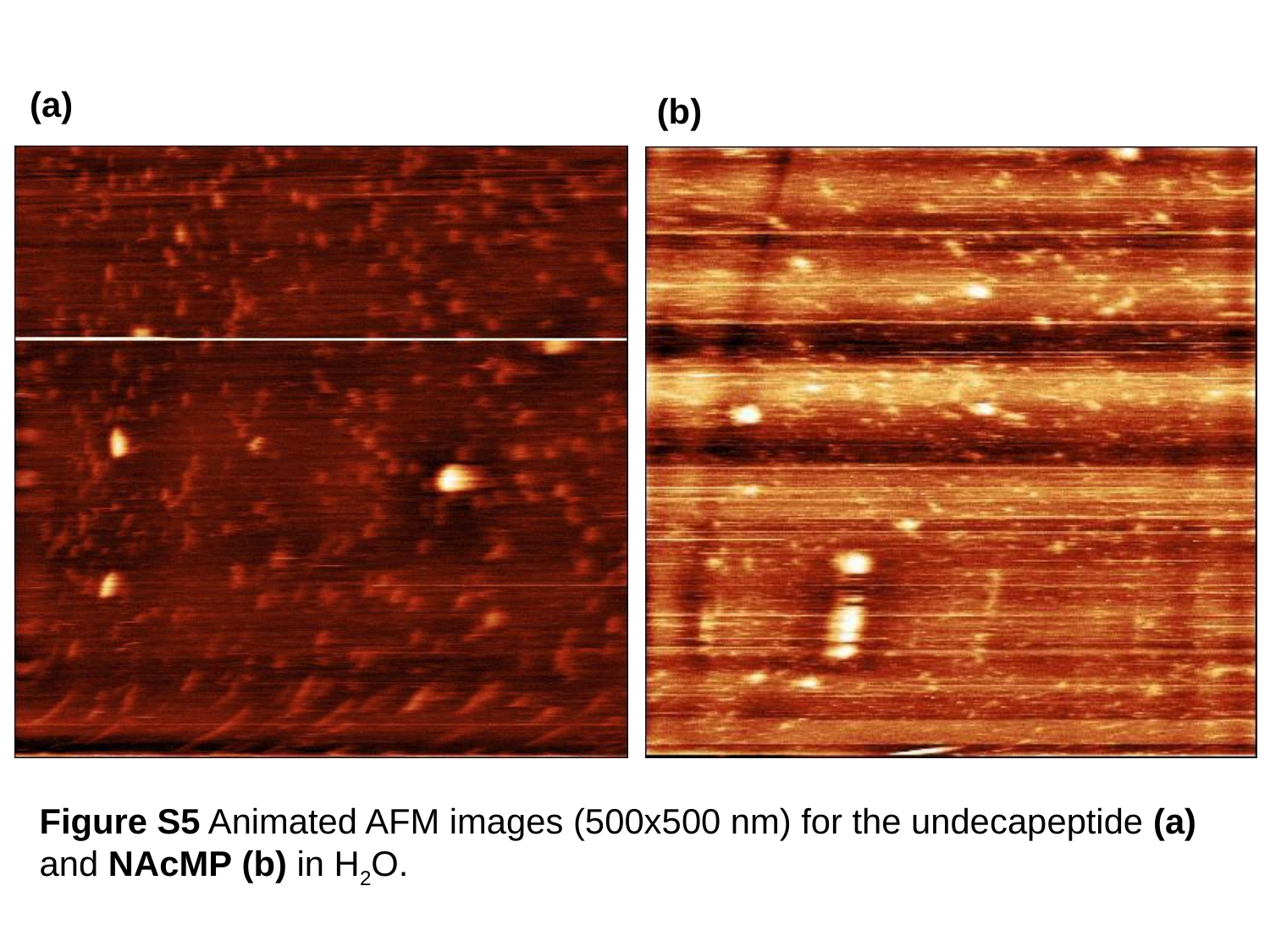

(a)
(b)
# Figure S5 Animated AFM images (500x500 nm) for the undecapeptide (a) and NAcMP (b) in H2O.

Supplement: nakanokoji-SI-AFM [file rsos172311supp2.pptx]
